# Functional high-throughput screening reveals miR-323a-5p and miR-342-5p as new tumor-suppressive microRNA for neuroblastoma

**DOI:** 10.1007/s00018-019-03041-4

**Published:** 2019-02-15

**Authors:** Aroa Soriano, Marc Masanas, Ariadna Boloix, Núria Masiá, Laia París-Coderch, Olga Piskareva, Carlos Jiménez, Kai-Oliver Henrich, Josep Roma, Frank Westermann, Raymond L. Stallings, Constantino Sábado, Josep Sánchez de Toledo, Anna Santamaria, Soledad Gallego, Miguel F. Segura

**Affiliations:** 1 Group of Translational Research in Child and Adolescent Cancer, Vall d’Hebron Research Institute (VHIR)-Universitat Autònoma de Barcelona (UAB), Passeig Vall d’Hebron 119-129, Collserola Building. Lab 207, 08035 Barcelona, Spain; 2 Institut de Ciència de Materials de Barcelona (ICMAB-CSIC) and Nanomol Technologies SA, Mòdul de Recerca B, Campus UAB, 08193 Bellaterra, Spain; 3 Cell Cycle and Cancer Laboratory, Biomedical Research Group in Urology, Vall d’Hebron Research Institute (VHIR)-Universitat Autònoma de Barcelona (UAB), Passeig Vall d’Hebron 119, 08035 Barcelona, Spain; 4 Molecular and Cellular Therapeutics, Royal College of Surgeons in Ireland and National Children’s Research Centre Our Lady’s Children’s Hospital, Dublin, Ireland; 5 Neuroblastoma Genomics Group, German Cancer Research Center (DKFZ), Im Neuenheimer Feld 280, 69120 Heidelberg, Germany; 6 Pediatric Oncology and Hematology Department, Hospital Universitari Vall d’Hebron-Universitat Autònoma de Barcelona (UAB), Passeig Vall d’Hebron 119, 08035 Barcelona, Spain

**Keywords:** Pediatric cancer, Non-coding RNA, High-throughput screening, 14q32, Epigenetics, Cancer therapy

## Abstract

Current therapies for most non-infectious diseases are directed at or affect functionality of the human translated genome, barely 2% of all genetic information. By contrast, the therapeutic potential of targeting the transcriptome, ~ 70% of the genome, remains largely unexplored. RNA therapeutics is an emerging field that widens the range of druggable targets and includes elements such as microRNA. Here, we sought to screen for microRNA with tumor-suppressive functions in neuroblastoma, an aggressive pediatric tumor of the sympathetic nervous system that requires the development of new therapies. We found miR-323a-5p and miR-342-5p to be capable of reducing cell proliferation in multiple neuroblastoma cell lines in vitro and in vivo, thereby providing a proof of concept for miRNA-based therapies for neuroblastoma. Furthermore, the combined inhibition of the direct identified targets such as CCND1, CHAF1A, INCENP and BCL-XL could reveal new vulnerabilities of high-risk neuroblastoma.

**Electronic supplementary material:**

The online version of this article (10.1007/s00018-019-03041-4) contains supplementary material, which is available to authorized users.

## Introduction

Approximately 15,000 new cases of pediatric cancers are diagnosed yearly in Europe, with around 10% corresponding to neuroblastoma (NB), an embryonal tumor of the sympathetic nervous system [1]. NB accounts for 15% of all cancer-related deaths in children, and is the embryonal tumor with the lowest 5-year relative survival [2]. Significant improvements are only foreseen in the field of new targeted therapies and personalized medicine programs which, at present, are effective for a small number of patients. Therefore, new approaches must be considered.

RNA therapeutics is an emerging field that widens the range of druggable targets and includes elements such as small interference RNA (i.e., siRNA, shRNA and microRNA). MicroRNA (miRNA) are small non-coding RNA that interfere with the translation and stability of coding mRNA in a sequence-specific manner [3]. Mounting evidence shows miRNA to be deregulated and functionally contributing to the development and progression of different human cancers, including NB [4, 5]. An overall reduction in miRNA is observed in advanced NB, mainly due to alterations in the miRNA-processing machinery [6]; therefore, miRNA restoration represents an attractive novel therapeutic approach.

To identify miRNA with therapeutic potential in NB, we carried out high-throughput functional screening of 2048 miRNA mimics. Several miRNA with potential tumor-suppressive functions were identified, among which miR-323a-5p and miR-342-5p had the highest therapeutic potential in multiple NB cell lines in vitro and in vivo. These results support the use of miRNA-based restoration therapies as an alternative tool against NB resistant to conventional therapies.

## Materials and methods

### Cell lines

SK-N-AS, SH-SY5Y, IMR-32 and HEK293T cell lines were purchased from American Type Culture Collection (ATCC, Manassas, VA, USA), CHLA-90 cell line from the Children’s Oncology Group Cell Culture and Xenograft Repository (Lubbock, TX, USA). SK-N-BE(2) and LA1-5s acquired from Public Health England Culture Collections (Salisbury, UK). All cell lines purchased from the tissue banks were amplified and stored in liquid nitrogen. Upon resuscitation, cells were maintained in culture for no more than 2 months. SK-N-AS, SK-N-BE(2), SH-SY5Y, IMR-32, CHLA-90 and LA1-5s were cultured and maintained in Iscove’s modified Dulbecco’s medium (Life Technologies, Thermo Fisher Scientific) supplemented with 10% heat-inactivated fetal bovine serum (South America Premium, Biowest) and 1% of insulin–transferrin–selenium Supplement (Life Technologies, Thermo Fisher Scientific). HEK293T were grown in Dulbecco’s modified Eagle’s medium (Life Technologies, Thermo Fisher Scientific) supplemented with 10% heat-inactivated fetal bovine serum. All media were supplemented with 100 U/mL penicillin, 100 µg/mL streptomycin (Life Technologies, Thermo Fisher Scientific) and 5 μg/mL plasmocin (InvivoGen). All cultures were maintained at 37 °C in a saturated atmosphere of 95% air and 5% CO_2_. All cell lines were frequently tested for mycoplasma contamination.

### MicroRNA functional high-throughput library screening

Chemoresistant SK-N-BE(2) cells were seeded in 96-well plates at 5000 cells/well using the MultidropV2 dispenser (Finstruments). Twenty-four hours later cells were transfected with a microRNA library consisting of 2048 human miRNA mimics (Dharmacon, Lafayette, CO, USA, miRIDIAN^®^ microRNA Library—Human Mimic (19.0) CS-001030 Lot 13112, GE Healthcare, 25 nM each miRNA) in technical triplicates using Lipofectamine 2000 (Life technologies, Thermo Fisher Scientific, Madrid, Spain, 0.2 µL per well) using the Robotic Platform Caliper Sciclone (Caliper Life Sciences, Perkin Elmer, Waltham, Massachusetts, USA). At 96-h post-transfection, cells were fixed with 1% glutaraldehyde (Sigma-Aldrich) and stained with 0.5% crystal violet (Sigma-Aldrich, Madrid, Spain). Crystals were dissolved with 15% acetic acid (Fisher Scientific) and absorbance was measured at 590 nm using an Epoch Microplate Spectrophotometer (Biotek, Winooski, Vermont, USA).

### Screening statistics

Data quality control and analysis of different factors (e.g., miRNA position, transfection time) was performed using “R” Statistical Software (Supplementary Fig. 1C).

The absorbance value of each mock- or miRNA-transfected well was normalized to the median of all non-transfected values of the corresponding replicate plate. To evaluate the effect of each miRNA on cell proliferation, the absorbance value (3 replicates) versus the median of all mock values was compared. The statistical significance was assessed by Student’s *t* test and the *p* value was adjusted by the false discovery rate “FDR” method. The percentage of proliferation was standardized using the *Z* score equation $Z = \frac{x - \mu }{\sigma }$, where *x* is the value of cell proliferation after transfection of each single miRNA, *µ* is the mean cell proliferation of all miRNA and *σ* the standard deviation.

### Mouse xenograft

SK-N-AS cells (4 × 10^6^) were reverse transfected with 25 nM of miR-control, miR-323a-5p or miR-342-5p in 100-mm plates (25 µL lipofectamine/dish). SK-N-BE(2) cells (4.7 × 10^6^) were reverse transfected with 25 nM of miR-control, miR-323a-5p or miR-342-5p in T175 flasks (90 µL lipofectamine/flask). Thirty-six hours post-transfection, 4 × 10^6^ cells/flank of SK-N-AS and 5 × 10^6^ cells/flank of SK-N-BE(2) were injected into the right flank of 6- to 8-week-old female NMRI-nude mice (*n* = 13 mice/condition of SK-N-AS and *n* = 15 mice/condition of SK-N-BE(2)) (Janvier Labs, Le Genest-Saint-Isle, France) in 300 µL of PBS:Matrigel (1:1). Tumor volume was measured every 2–3 days. At the end of the experiment, the primary tumors were removed and weighted. Part of the tumors were fresh frozen and the rest fixed in 10% formalin and embedded in paraffin.

### Statistical methods

Unless otherwise stated, mean ± SEM values are representative of the average of three independent experiments. Statistical significance was determined by unpaired two-tailed Student’s *t* test (GraphPad Prism Software, La Jolla, CA, USA.). * Means *p* < 0.05, ** means *p* < 0.01 and *** means *p* < 0.001.

## Results

### Functional high-throughput miRNA screening identified several miRNA with tumor-suppressive activity

**Fig. 1:**
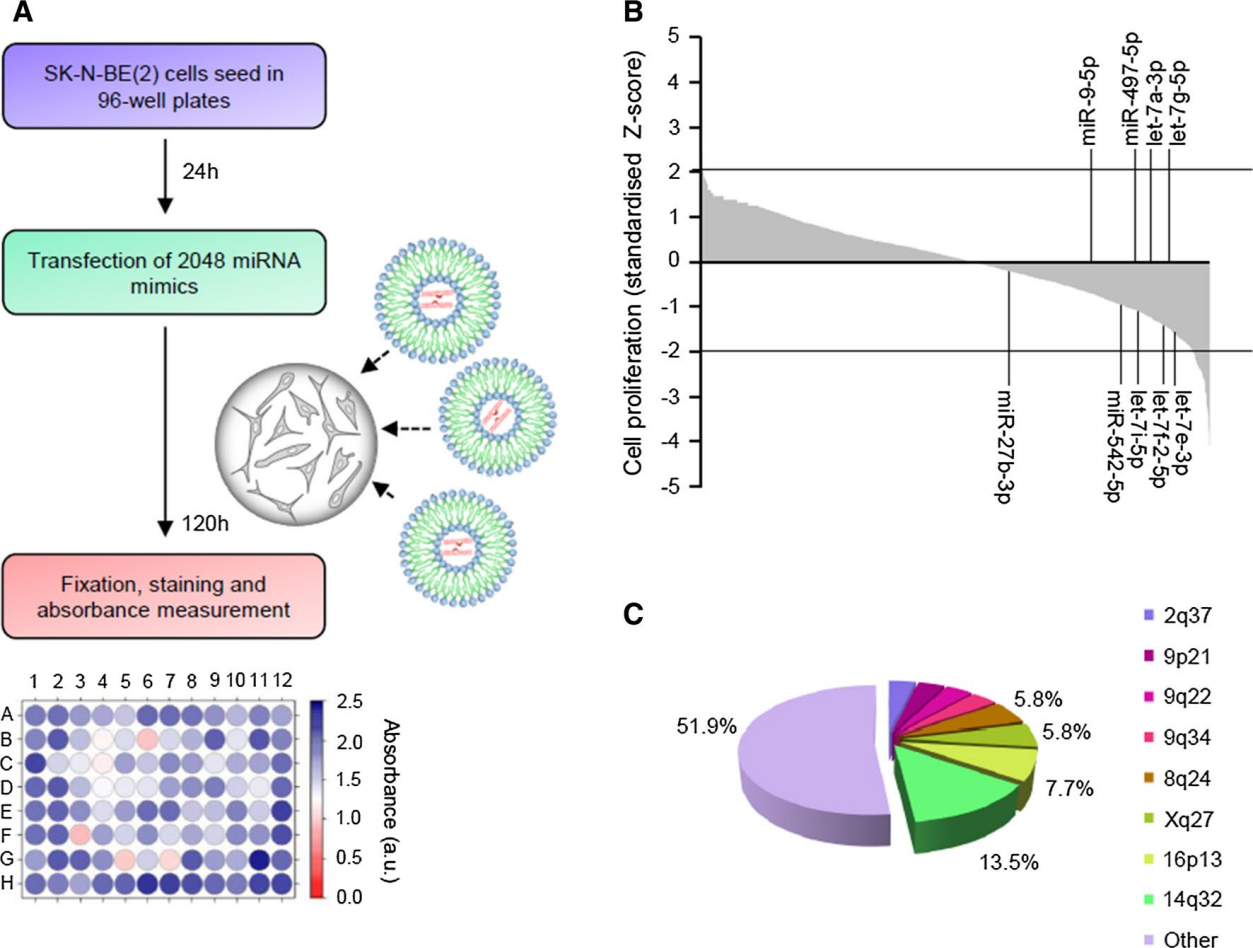
Functional high-throughput miRNA screening identified several tumor-suppressive miRNA. **a** Screening design. **b** Graph representing the effects of 2048 individual miRNA on cell proliferation. The percentage of growth inhibition was obtained by comparing the average of three independent miRNA mimics replicates with mock-transfected cells and standardized using the *Z* score transformation method. MiRNA previously reported as tumor-suppressive miRNA in NB are indicated. **c** Pie chart representing the genomic distribution of miRNA that were capable of reducing cell proliferation ~ 50% (*Z* score < − 2)

High-throughput screening using the largest library of miRNA mimics available was performed to identify miRNA with tumor-suppressive functions in NB. Individual miRNA mimics were transfected into the SK-N-BE(2) cells (Fig. 1a). Control miRNA (i.e., cel-miR-67 and cel-miR-239b) were used as negative controls and miR-497-5p as a positive control [7, 8] (Supplementary Fig. 1a,b). The overexpression of 52 miRNA was found to reduce ~ 50% cell proliferation (*Z* score < − 2, adjusted *p* value < 0.05) (Fig. 1b; Supplementary Tables 1, 2).

Since it has been suggested that miRNA with similar functions cluster together [9], we examined the genomic distribution of miRNA whose overexpression produced the highest reduction on cell proliferation. Interestingly, 7 of the 52 miRNA (13.5%) were located at 14q32 (Fig. 1c and Supplementary Table 2), a locus with high miRNA density and frequently lost or silenced in different types of tumors, including NB [10].

### Restoration of miRNA located at 14q32 reduced cell viability in multiple NB cell lines

**Fig. 2:**
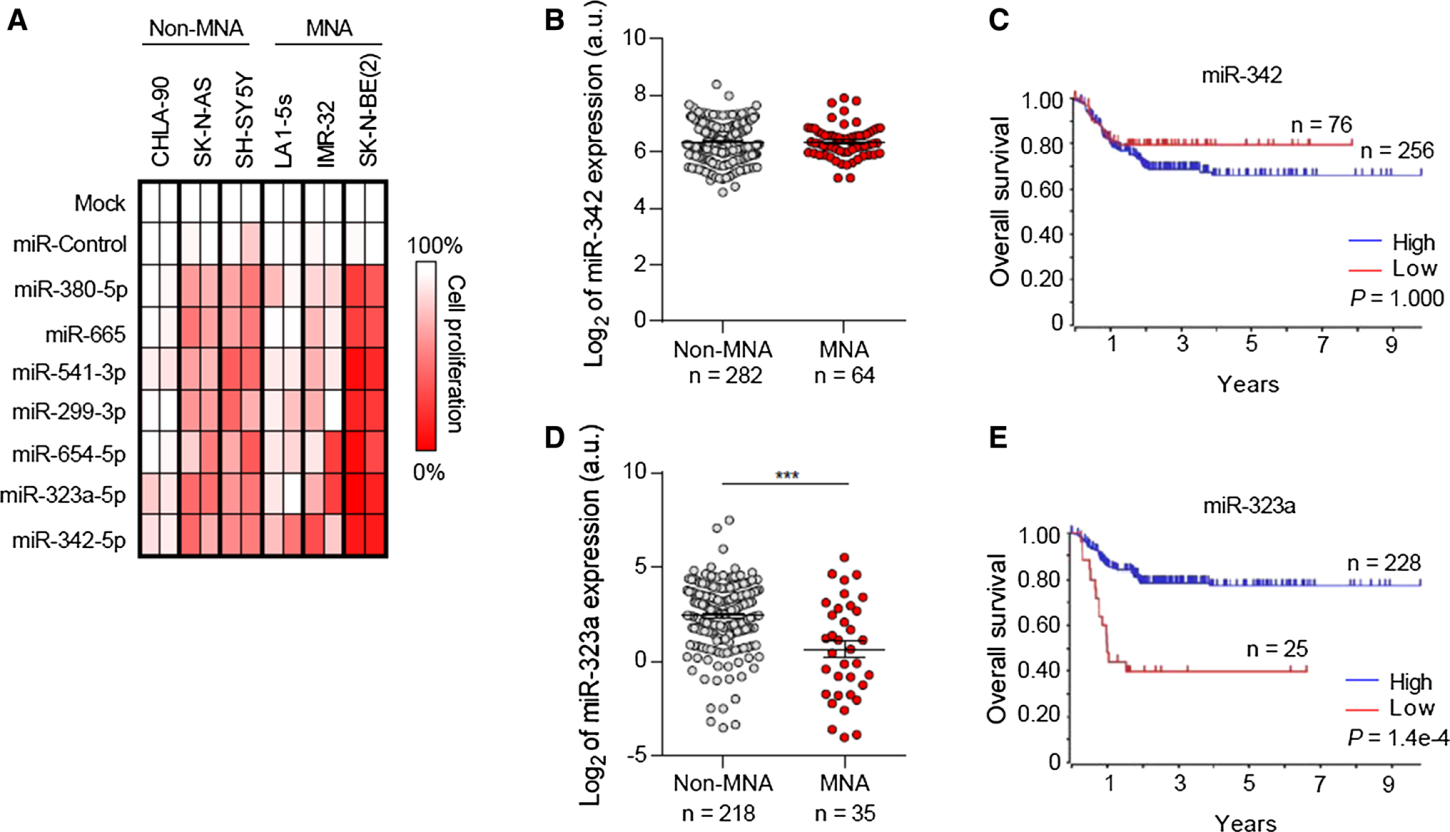
Ectopic expression of miRNA located at 14q32 reduced cell proliferation in multiple NB cell lines. **a** Heatmap representing effects of the indicated miRNA on cell proliferation reduction in MYCN amplified (MNA) and MYCN non-amplified (non-MNA) cell lines. The overexpression effect of each miRNA on cell proliferation was compared to mock-transfected cells. **b**, **d** Relative expression (log_2_) of miR-342 (**b**) and miR-323a (**d**) in MNA and non-MNA NB tumors. **c**, **e** Overall survival Kaplan–Meier plot of the indicated miRNA expression in human NB tissues

The tumor-suppressive effects of miRNA hits located at 14q32 (i.e., miR-380-5p, miR-665, miR-541-3p, miR-299-3p, miR-654-5p, miR-323a-5p and miR-342-5p) were further confirmed in an extended panel of six NB cell lines bearing genomic alterations associated with resistance to standard NB therapies and poor patient outcome (Supplementary Table 3). The overexpression of all miRNA tested reduced cell proliferation in multiple NB cell lines compared with mock- and miR-control-transfected cells being miR-323a-5p and miR-342-5p those ones with the highest therapeutic potential (Fig. 2a).

### Low miR-323a-5p expression levels correlate with poor patient outcome

The expression levels of miR-323a-5p and miR-342-5p were analyzed in a large cohort of NB tissue samples and correlated with clinical parameters. Whereas lower expression levels of miR-342-5p did not correlate with the clinical parameters analyzed (Fig. 2b–c), low miR-323a-5p levels were found to correlate with MYCN genomic amplification (Fig. 2d) and shorter overall survival (Fig. 2e).

### Ectopic expression of miR-323a-5p and miR-342-5p halted cell cycle progression and induced apoptosis in NB

**Fig. 3:**
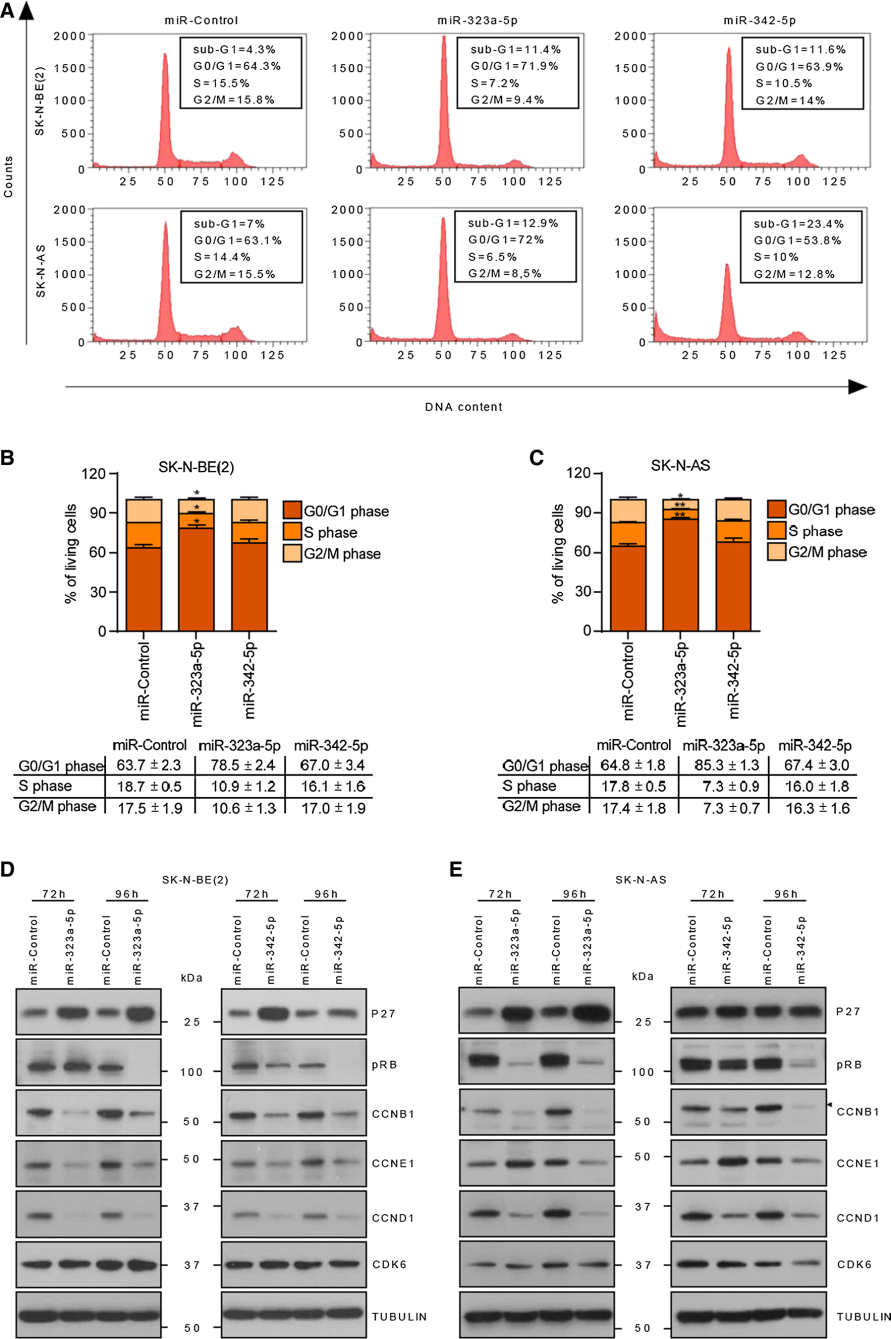
MiR-323a-5p induced cell cycle arrest at G0/G1 phase. Cell cycle flow cytometry analysis of the indicated cell lines transfected with miR-control, miR-323a-5p or miR-342-5p at 25 nM for 96 h. **a** One representative histogram of three independent experiments. **b**, **c** Histograms representing the average percentage of living cell population from three independent experiments of SK-N-BE(2) (**b**) or SK-N-AS (**c**) cells in G0/G1, S, or G2/M phases. *p < 0.05, **p < 0.01, two-tailed Student’s *t* test. **d**, **e** Western blot of the indicated cell cycle regulatory proteins in SK-N-BE(2) (**d**) and SK-N-AS (**e**) transfected with miR-control, miR-323a-5p and miR-342-5p (25 nM) at 72 h and 96 h post-transfection

To clarify whether the reduction in cell number upon miR-323a-5p and miR-342-5p overexpression was due to inhibition of cell proliferation and/or induction of cell death, cell cycle progression was analyzed using flow cytometry. The overexpression of miR-323a-5p induced a modest increase in the subG1 peak and an increment in G0/G1-phase population accompanied by a reduction in the S and G2/M phases. Therefore, both cell cycle arrest and increased cell death might be contributing to the miR-323a-5p overexpression phenotype (Fig. 3a–c). Concurring with these observations, a reduction in cyclins, D1, E1 and B1 was observed after miR-323a-5p overexpression at 72 h post-transfection and a consequent reduction in phospho-RB levels at later time points. Furthermore, upregulation of the cell cycle inhibitor p27 was also observed (Fig. 3d, e).

MiR-342-5p-transfected cells showed no apparent differences regarding cell cycle progression compared to miR-control cells, but did show an accumulation of cells in subG1, thereby indicating cell death induction (Fig. 3a–c). In this case, reduced levels of cyclins, D1, E1 and B1 and phospho-RB, but not increased p27 levels, were also observed (Fig. 3d, e), suggesting that cells committed to die were unable to progress through the cell cycle.

**Fig. 4:**
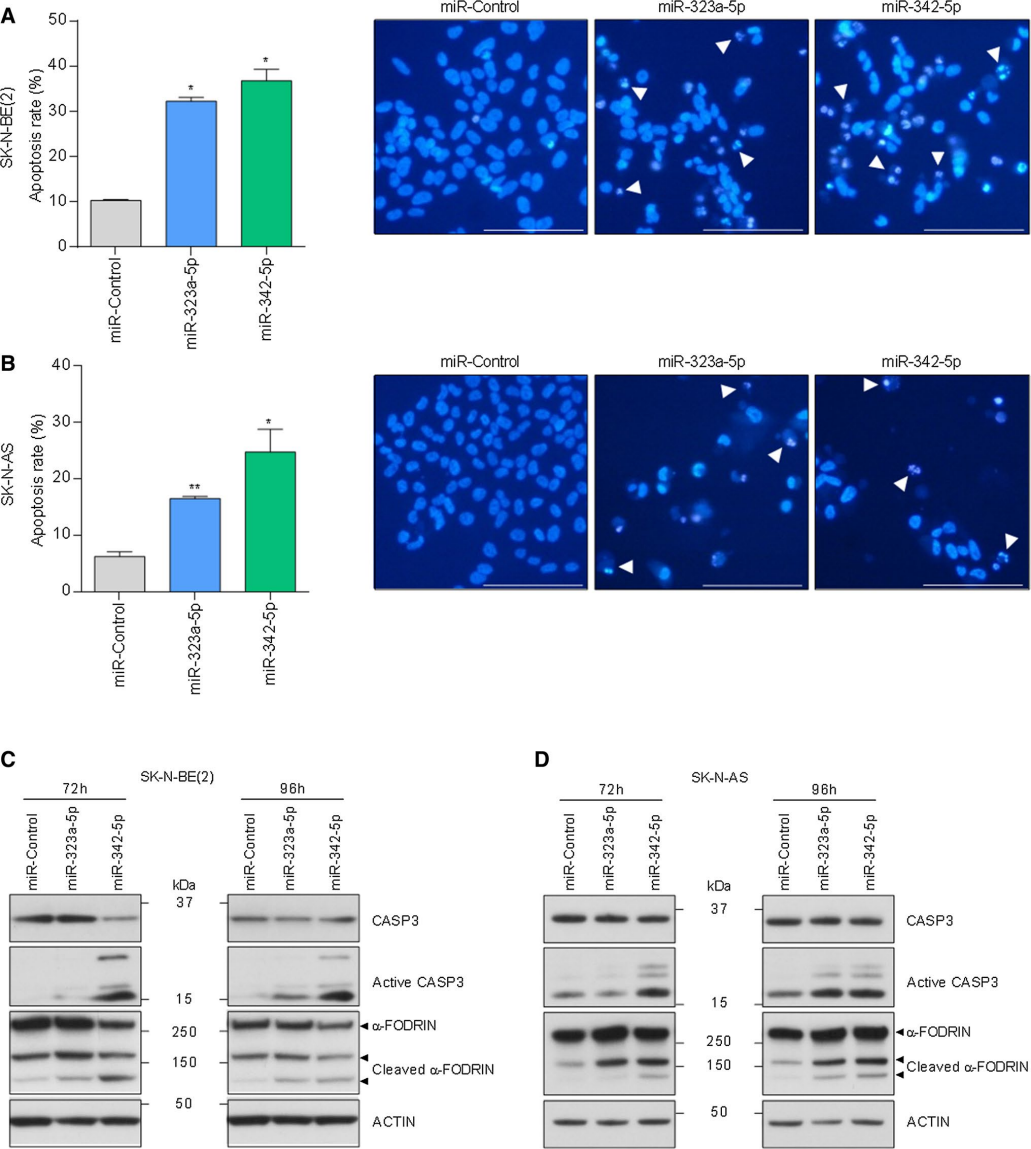
MiR-323a-5p and miR-342-5p overexpression induced apoptosis in NB cells. **a**, **b** Analysis of chromatin fragmentation/condensation in SK-N-BE(2) (**a**) and SK-N-AS (**b**) transfected with 25 nM of miR-control, miR-323a-5p or miR-342-5p 96 h post-transfection. Images on the right show a representative field of NB cells stained with Hoechst dye. White arrowheads point to cells with condensed and/or fragmented chromatin. Data represent mean ± SEM of three independent experiments (*n* = 3 per experiment). *p < 0.05, **p < 0.01, two-tailed Student’s *t* test. **c**, **d** Representative Western blot analysis of apoptosis-related proteins in SK-N-BE(2) (**c**) and SK-N-AS (**d**) transfected with miR-control, miR-323a-5p or miR-342-5p (25 nM) at 72 h and 96 h post-transfection

Next, we examined the chromatin status of miRNA-transfected cells by Hoechst staining. MiR-323a-5p and miR-342-5p transfected cells showed a higher percentage of cells with condensed and fragmented chromatin staining, one of the typical hallmarks of apoptosis (Fig. 4a, b). Furthermore, NB cells transfected with miR-323a-5p and miR-342-5p showed cleavage of the executor caspase-3 and one of its targets α-FODRIN, both indicative of apoptotic cell death (Fig. 4c, d).

### MiR-323a-5p and miR-342-5p target cell cycle and survival genes

**Fig. 5:**
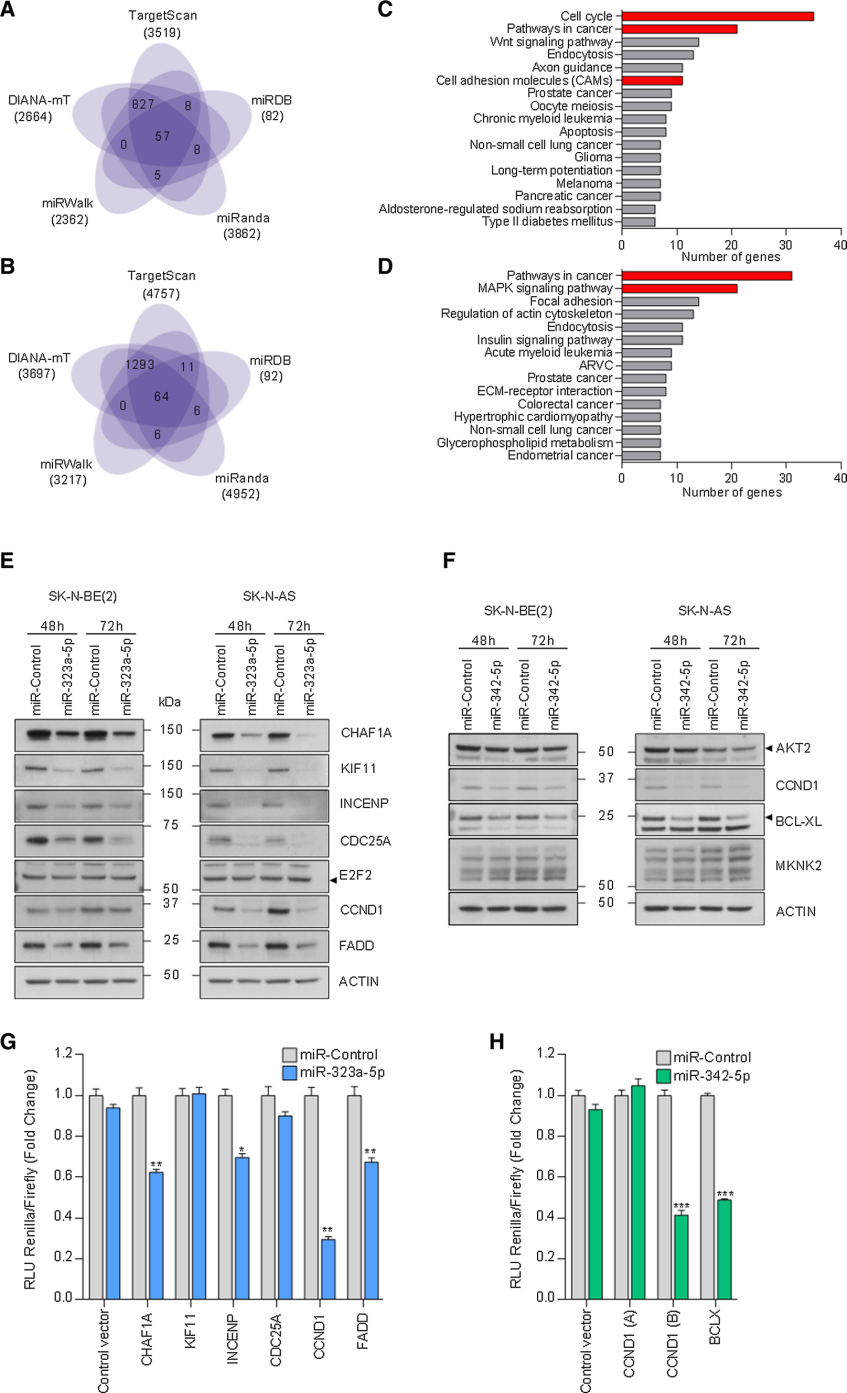
MiR-323a-5p and miR**-**342-5p modulated the expression of multiple cancer-related genes. **a**, **b** Venn diagram representing the overlap of predicted target genes among five miRNA-target prediction algorithms of miR-323a-5p or miR-342-5p. **c**, **d** Representation of the functional annotation of predicted target genes of miR-323a-5p (**c**) or miR-342-5p (**d**) using KEGG pathways and Gene Ontology databases. Red bars indicate selected pathways to analyze potential miRNA targets. **e**, **f** Representative Western blot of predicted target genes in SK-N-BE(2) and SK-N-AS transfected with miR-control, miR-323a-5p (**e**) or miR-342-5p (**f**) at 48 h and 72 h post-transfection. **g**, **h** Luciferase 3′UTR reporter assays. Graph represents luciferase activity in HEK-293T cells co-transfected with 50 ng/well of the indicated reporter vectors and 25 nM of miR-control, miR-323a-5p (**g**) or miR-342-5p (**h**). Data represented the average ± SEM of three independent experiments (*n* = 3 per experiment). *p < 0.05, **p < 0.01, ***p < 0.001

To find the downstream mediators of miR-323a-5p and miR-342-5p phenotypic effects in NB, a miRNA-target analysis was performed comparing five different platforms (Fig. 5a, b). For miR-323a-5p, 905 potential targets were predicted by at least four independent algorithms, whereas 1380 were predicted for miR-342-5p (Supplementary Tables 4, 5). According to the Kyoto Encyclopedia of Genes and Genomes (KEGG) pathway analysis [11], a significant number of these predicted targets were associated with cell cycle or commonly altered pathways in cancer (Fig. 5c, d, *p* value < 0.05). These genes were selected for validation based on their expression association with NB outcome and/or previous reported functional role in cancer (Supplementary Tables 6, 7). Transient miRNA overexpression proved to consistently reduce the mRNA levels of several miR-323a-5p (i.e., *CHAF1A*, *KIF11*, *INCENP*, *CDC25A*, *CCND1*, *FADD* and *E2F2*) and miR-342-5p (i.e., *AKT2*, *CCND1*, *MKNK2* and *BCLX*) predicted targets (Supplementary Fig. 2a–d). To confirm the reduction also in protein levels, Western blot was performed at 48 h and 72 h post-transfection of miR-323a-5p or miR-342-5p. CHAF1A, KIF11, INCENP, CDC25A, CCND1 and FADD protein levels decreased in both cell lines when cells were transfected with miR-323a-5p (Fig. 5e). On the other hand, only CCND1 and BCL-XL protein levels were reduced in both cell lines when cells were transfected with miR-342-5p, whereas no differences were found in AKT2 levels. Results for MKNK2 were not conclusive (Fig. 5f).

To ascertain whether the potential targets were regulated directly by miR-323a-5p or miR-342-5p, we engineered luciferase-reporter vectors with the 3′UTR of genes bearing the putative miRNA-binding sites (Supplementary Fig. 3). The reporter vectors were co-transfected with a miR-control, miR-323a-5p or miR-342-5p and luciferase activity was quantified 24 h after transfection. The overexpression of miR-323a-5p caused a reduction in luciferase activity in the *CHAF1A*, *INCENP*, *CCND1* and *FADD* 3′UTR reporter vectors and no differences were observed for *KIF11* and *CDC25A*, thereby indicating that these last two genes are not direct targets (Fig. 5g). On the other hand, reduction in the luciferase activity of *CCND1* and *BCLX* 3′UTR vectors by miR-342-5p overexpression confirmed both genes as direct miR-342-5p targets (Fig. 5h).

### CCND1, CHAF1A and INCENP silencing mostly reproduced miR-323a-5p overexpression effects

**Fig. 6:**
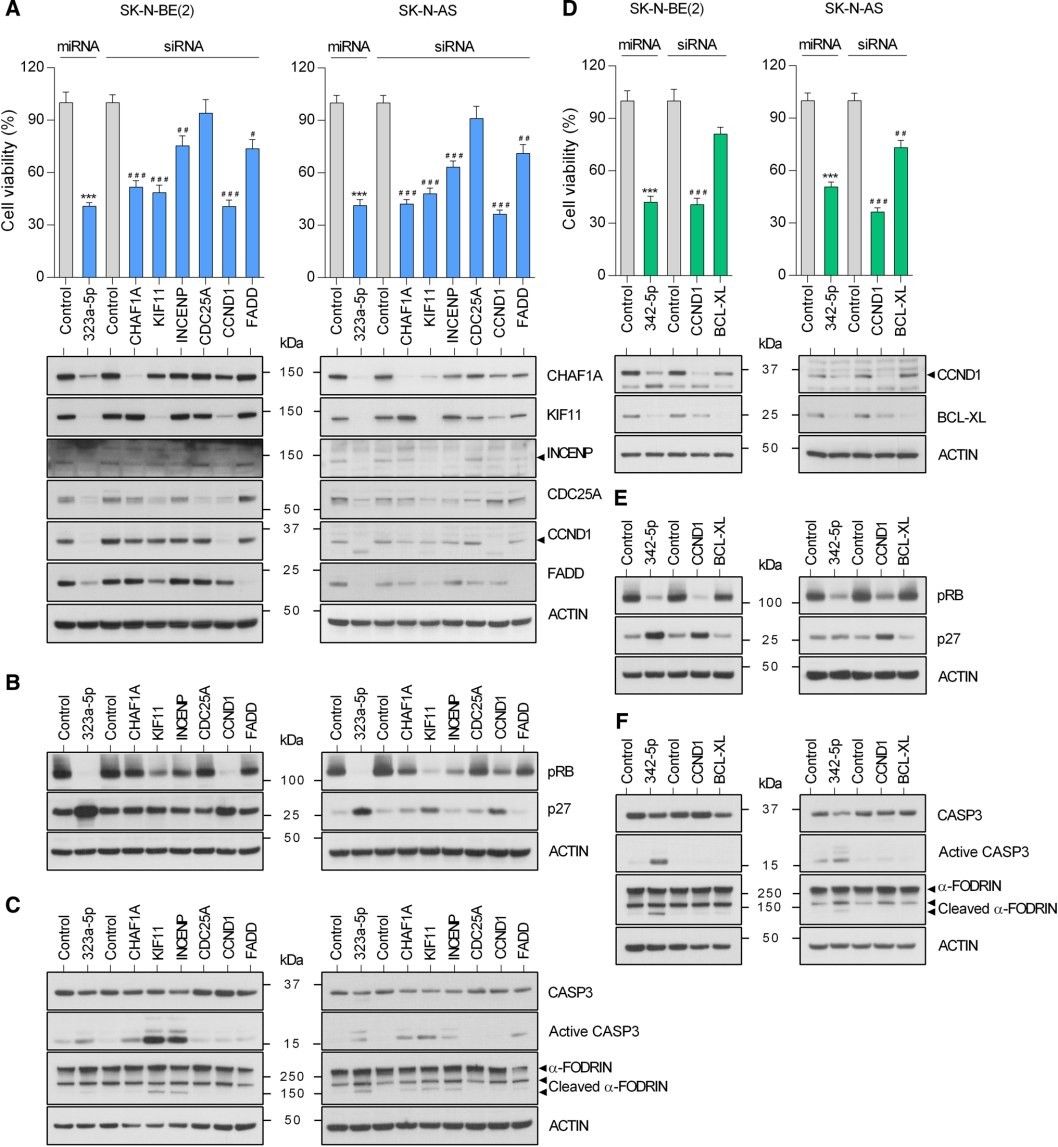
MiRNA target-knockdown partially reproduced the anti-tumoral effects of miR-323a-5p and miR-342-5p. **a**, **d** Cell viability assay of SK-N-BE(2) and SK-N-AS transfected with 25 nM of miR-323a-5p, miR-342-5p or the indicated siRNA. Target protein knockdown was analyzed by Western blot at 72 h post-transfection (lower panels). **b**, **e** Expression of some representative cell cycle regulatory proteins in SK-N-BE(2) and SK-N-AS transfected with miR-323a-5p, miR-342-5p or the indicated siRNA (25 nM) at 96 h post-transfection. **c**, **f** Western blot analysis of apoptosis-related proteins in SK-N-BE(2) and SK-N-AS transfected with miR-323a-5p, miR-342-5p or the indicated siRNA (25 nM) at 96 h post-transfection. Graph represents one of three independent experiments (*n* = 6 per experiment). Asterisk compares miR-323a-5p or miR-342-5p versus miR-Control and hash compares each siRNA versus control siRNA. * or ^#^*p *< 0.05, ** or ^##^*p *< 0.01, *** or ^###^*p *< 0.001, two-tailed Student’s *t* test

The contribution of each target to the miRNA overexpression phenotype was analyzed by siRNA-mediated gene silencing. Silencing of CHAF1A, KIF11 and CCND1 showed a reduction in cell proliferation similar to that induced by miR-323a-5p, while INCENP and FADD silencing only produced a moderate effect (Fig. 6a). Western blot analysis 72 h post-transfection confirmed siRNA efficacy (Fig. 6a, lower panels). Overall, CCND1 depletion mirrored the best miR-323a-5p overexpression, not only the general effects on cell proliferation, but also on the reduction in phospho-RB levels and p27 accumulation (Fig. 6b). However, when apoptosis induction was analyzed, CHAF1A and INCENP were the direct targets that better phenocopied the effects of miR-323a-5p overexpression (Fig. 6c). These results suggest that the combination of CCND1, CHAF1A and INCENP inhibition is sufficient to reproduce the therapeutic effects of miR-323a-5p.

### Inhibition of CCND1 and BCL-XL partially reproduces the effects of miR-342-5p

The same strategy was used to characterize the contribution of miR-342-5p targets. In this case, only the inhibition of CCND1 showed a similar reduction in cell proliferation (Fig. 6d). In agreement with this observation, only CCND1 silencing caused a reduction in phospho-RB levels and accumulation of the cell cycle inhibitor p27 (Fig. 6e). However, apoptosis induction was not observed with silencing of either CCND1 or BCL-XL (Fig. 6f) suggesting that the observed effects of miR-342-5p on cell death might require the inhibition of both or additional targets.

### MiR-323a-5p and miR-342-5p overexpression reduced tumor growth in vivo

**Fig. 7:**
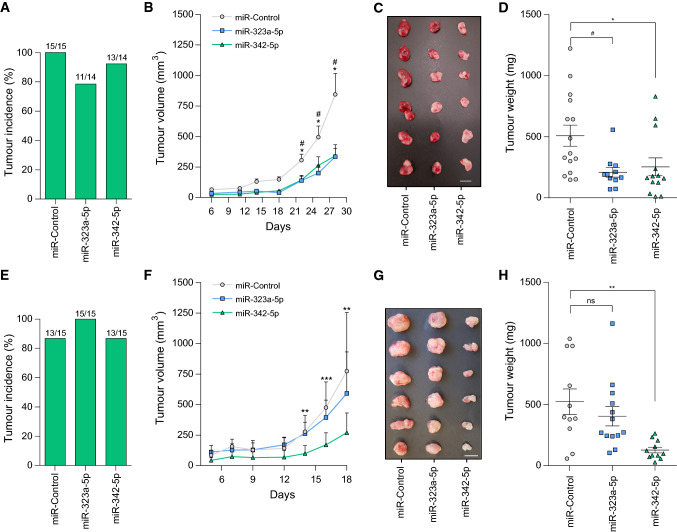
MiR-323a-5p and MiR-342-5p overexpression reduced tumor growth in vivo. **a**, **e** Tumor formation incidence in SK-N-BE(2) (**a**) and SK-N-AS (**e**). **b**, **f** Tumor growth of xenografts derived from SK-N-BE(2) (**b**) SK-N-AS (**f**) cells transfected with miR-control, miR-323a-5p or miR-342-5p. Tumor measurements were taken three times per week for ~ 3 weeks. **c**, **g** Representative images of dissected tumors (bar indicates 1 cm). **d**, **h** Tumor weight at 18 days post-injection. Hash compares miR-323a-5p versus control and asterisk compares miR-342-5p versus control. * or ^#^*p *< 0.05, **p < 0.01, ***p < 0.001, two-tailed Student’s *t* test

We proceeded to validate the capacity of miR-323a-5p and miR-342-5p to reduce tumor growth in vivo by subcutaneous injection of SK-N-BE(2) or SK-N-AS cells previously transfected with miR-control, miR-323a-5p or miR-342-5p into the flank of NRMI-nude mice. At the end of the experiment, all groups showed a similar percentage of tumor incidence (~ 80–100%, Fig. 7a, e), which suggested that miRNA overexpression did not interfere with tumor engraftment. Tumor growth was monitored every 2–3 days for 28 days for SK-N-BE(2) or 18 days for SK-N-AS. While in SK-N-AS, only the overexpression of miR-342-5p was capable of delaying cell growth in vivo, both miRNA were effective in SK-N-BE(2) cells, showing a final ~ twofold reduction in tumor growth at the end of the experiment (Fig. 7b, f). These effects were confirmed by the analysis of tumor weight (Fig. 7c, d, g, h-Supplementary Fig. 4a, b).

In summary, our results confirm miR-323a-5p and miR-342-5p as potential therapeutic tools against high-risk NB. Of note, miR-323a-5p overexpression impaired tumor growth in the MYCN-amplified cell line, but not in SK-N-AS, suggesting that higher doses of miR-323a-5p or being functional for longer periods of time may be needed to achieve a therapeutic effect. In support of this hypothesis, miRNA levels were analyzed at the beginning and at the end of experiment. Both cell lines achieved similar levels of miRNA overexpression at the time of injection (Supplementary Fig. 5a, c) ruling out the possibility of differential transfection efficiency between cell lines. However, at the end of experiment, SK-N-AS maintained much lower levels of miR-323a-5p in SK-N-AS but not in SK-N-BE(2) (Supplementary Fig. 5b, d), thus suggesting that this miRNA is processed faster in this cell line and therefore, tumor cells overcome more easily the miR-323a-5p tumor-suppressive effects.

## Discussion

Despite the emerging personalized medicine programs for pediatric tumors, which include molecular profiling of NB, the small number of recurrent somatic mutations found at diagnosis and the lack of established tumor drivers, limit the therapeutic opportunities for NB patients. On the other hand, some patients relapse and develop drug resistance when treated with anticancer drugs targeting single molecules, which might be due to the activation of alternative pathways.

MiRNA alterations have been shown to participate in the progression and outcome of NB (reviewed in [12]). We propose the use of miRNA as multi-target approach to overcome treatment resistance in NB patients, given that a single miRNA can inhibit multiple targets involved in tumorigenic processes.

Multiple pharmaceutical companies have already included miRNA in their developmental drug pipelines (e.g., miR-34 [13, 14], miR-16 [15], miR-122 [16]). The first challenge in applying this approach to the treatment of high-risk NB is to identify those miRNA with the highest therapeutic potential. Our work is the largest high-throughput functional screening performed to date to identify miRNA with tumor-suppressive functions in cancer and particularly for NB. Of the 2048 miRNA tested, we identified 52 that significantly reduced cell proliferation with a *Z* score lower than − 2 (~ 50% of cell proliferation). Concurring with previous reports, multiple tumor-suppressive miRNA such as members of the let-7 miRNA tumor-suppressive family (e.g., let-7i-5p, let-7a-3p, let-7f-2-5p, let-7g-5p or let-7e-3p [17, 18]) were also identified in our screening, thereby validating our approach to identifying tumor-suppressive miRNA.

Notably, 7 of the 52 miRNA (13.5% of the total) shown to reduce cell proliferation ~ 50% are encoded in the 14q32 genomic region. This region is frequently altered in cancer and particularly in NB [10], which raises the possibility that this region contains tumor-suppressor genes. All 7 miRNA identified in our screening showed the capacity to reduce cell proliferation in multiple NB cell lines in vitro, with miR-323a-5p and miR-342-5p being those with the highest therapeutic potential.

In agreement with our results, miR-323a-5p has been reported to be downregulated in NB patients with MYCN amplification and associated with unfavorable outcome [19, 20]. Moreover, miR-323a-5p overexpression has been reported to reduce proliferation and promote apoptosis of human cerebral glioma cells [21]. We found that ectopic expression of miR-323a-5p suppressed cell growth through the direct modulation of several cell cycle-associated genes such as *CCND1*, *CHAF1A*, *INCENP* and *FADD* and caused a G1 cell cycle arrest followed by induction of apoptosis. The silencing of the miR-323a-5p direct targets CHAF1A, INCENP and CCND1 were those that more closely recapitulate the phenotypic effects of miR-323a-5p overexpression. CCND1 is a well-established oncogene frequently overexpressed and associated with poor outcome in different types of tumors including NB [25]. CCND1 interacts with CDK4 and CDK6 and the activation of this complex phosphorylates RB and other transcription factors which promote cell cycle progression [26]. To date, the best approach to targeting CCND1 is through the use of CDK4/6 inhibitors such as palbociclib, ribociclib and abemaciclib.

Although the best known functions of CCND1 are related to cell cycle control, CCND1 has CDK-independent functions. For example, CCND1 regulates cell differentiation by binding to several transcription factors such as the estrogen receptor α (ERα) and the androgen receptor (AR) (reviewed in [27]). Therefore, a direct inhibition of CCND1 could be therapeutically more effective than CDK4/6 inhibition.

Another of the newly identified relevant miR-323a-5p target is CHAF1A, a chromatin modifier protein recently involved in maintaining the undifferentiated state of highly aggressive NB [28]. Thus, in addition to halting cell cycle progression, miR-323a-5p could also be relevant for NB therapy through repressing the expression of genes that block the differentiation of NB cells.

The silencing of INCENP phenocopied the apoptosis induction of miR-323a-5p overexpression. INCENP (inner centromere protein) is a component of the chromosomal passenger complex (CPC), a complex that regulates mitosis. INCENP is a scaffolding subunit for the CPC and activates Aurora B kinase [29]. Similar to *CHAF1A*, high levels of *INCENP* in primary NB tumors are associated with poor prognosis (Supplementary Table 6). In addition, silencing of INCENP using doxycycline-inducible shRNA led to significant decreases in growth of NB xenografts and increases mice survival (Sun et al. Advances in Neuroblastoma Research 2018). Therefore, targeting INCENP could be a novel promising therapy in NB.

Our in vivo studies suggested that the transient overexpression of miR-323a-5p was enough to halt the proliferation of MYCN-amplified NB cells but not in SK-N-AS xenograft models, probably due to a higher rate of miRNA processing. At that point, and owing to technical limitations, the fact that more sustained overexpression of miR-323a-5p could have a better therapeutic response cannot be ruled out.

In contrast, the transient overexpression of miR-342-5p did show faster induction of cell death in vitro and a clear reduction in tumor growth in vivo in both NB xenografts tested. In line with our observations, miR-342-5p was reported to be downregulated in breast cancer patients with early relapse [22] and was able to reduce HER2-positive breast cancer cell growth [23] and colon cancer cells [24].

Interestingly, we found that miR-342-5p is also a direct modulator of CCND1. CCND1 silencing alone caused a dramatic decrease in NB cell proliferation, but was not enough to induce cell death (Fig. 6c, f). We also identified BCL-XL as a direct target of miR-342-5p. BCL-XL is an anti-apoptotic member of the B cell lymphoma 2 (BCL-2) protein family whose overexpression contributes to tumor progression and resistance to chemotherapeutic agents [30]. Although BCL-XL reduction alone only causes a minimal reduction in cell number and did not induce cell death (Fig. 6f), it may lower the threshold of apoptosis induction upon CCND1 silencing or inhibition. Therefore, the concomitant reduction in CCND1 and BCL-XL would more faithfully reproduce the overexpression effects of miR-342-5p.

## Conclusions

Our strategy is confirmed as valid for the identification of novel tumor-suppressive miRNA such as miR-323a-5p and miR-342-5p in NB models, including but not limited to the ones that are resistant to conventional therapies and reveals new vulnerabilities of high-risk NB through the combined inhibition of targets such as CCND1, CHAF1A, INCENP and BCL-XL.

### Electronic supplementary material

Below is the link to the electronic supplementary material.

Supplementary material 1 (DOCX 675 kb)

Supplementary material 2 (XLSX 447 kb)

Supplementary material 3 (DOCX 18 kb)
