# Supplementary material for: Functional high-throughput screening reveals miR-323a-5p and miR-342-5p as new tumor-suppressive microRNA for neuroblastoma
Source: Cell Mol Life Sci. 2019 Feb 15;76(11):2231–43. doi: 10.1007/s00018-019-03041-4 (PMC6502783; doi:10.1007/s00018-019-03041-4)

**SUPPLEMENTARY METHODS**

**Analysis of miRNA expression in human samples**

MiRNA expression data from NB tumors was obtained from the Tumor Neuroblastoma Compendium (NRC) dataset. A total of 365 NB samples were obtained and analyzed from patients enrolled by Our Lady’s Hospital for Sick Children (Crumlin, Dublin, Ireland), the Children’s Oncology Group (Philadelphia, USA), the Ghent University Hospital (Ghent, Belgium), the Academic Medical Center (AMC; Amsterdam, Netherlands) and the University Children’s Hospital Essen (Essen, Germany). Tumors were profiled for 430 miRNA plus 36 control small RNA using individual Taqman PCR assays setup in 384-well format and mRNA gene expression profile was performed by Affymetrix GeneChip HG-U133plus2.0. Patient characteristics are listed in and data set has been previously described in a number of publications[1].

**Cell proliferation assays**

Cells were seeded in 96-well plates at the following densities: LA1-5s, 1.5 x 10^3^ cells/well; SK-N-AS, 4 x 10^3^ cells/well; CHLA-90 8 x 10^3^ cells/well; IMR-32, SK-N-BE(2) and SH-SY5Y at 5 x 10^3^ cells/well. Twenty-four hours later, cells were transfected with the indicated miRIDIAN microRNA mimic oligonucleotides (25 nM). For siRNA cell viability assay, SK-N-BE(2) and SK-N-AS (9 x 10^3^ and 7 x 10^3^ cells/well, respectively) were seeded in 96-well plates (6 replicates/condition). Cells were reverse transfected with non-targeting-BLOCK-iT siRNA control (Thermo Fisher Scientific) or with the indicated siRNA oligonucleotides (Sigma, 25 nM, Supplementary Table 8). At 96 h post-transfection, cells were fixed with 1% glutaraldehyde (Sigma-Aldrich) and stained with 0.5% crystal violet (Sigma-Aldrich). Crystals were dissolved with 15% acetic acid (Fisher Scientific) and optical density was read at 590 nm using an Epoch Microplate Spectrophotometer (Biotek).

**Cell cycle analysis**

SK-N-AS (1.3 x 10^6^) and SK-N-BE(2) (1.4 x 10^6^) cells were reverse transfected with 25 nM of miR-control, miR-323a-5p mimic or miR-342-5p mimic in 100-mm dishes (25μL of Lipofectamine/dish). After 96 h, cells were harvested and fixed with cold 70% ethanol and kept at 4ºC for at least 24 h. Prior to flow cytometry analyses, cells were washed twice in PBS and resuspended in staining solution [0.19 mM sodium citrate (Sigma-Aldrich), 500 μg/mL propidium iodide (Thermo Fisher Scientific) and 10 mg/mL RNAse DNAse-free (AppliChem)] and incubated overnight. Analysis of DNA content was analyzed using a FACScalibur flow cytometer (BD Biosciences) and data by BD CellQuest^TM^ Pro Software (BD Biosciences).

**Cell death assay**

SK-N-AS (4.5 x 10^4^) and SK-N-BE(2) (6 x 10^4^) cells were seeded in 24-well plates and reverse transfected with Lipofectamine 2000 with 25 nM of miR-control, miR-323a-5p mimic or miR-342-5p mimic in triplicate. At 96 h post-transfection, cells were stained with 0.05 µg/ml Hoechst 33258 dye and photographed. Apoptosis quantification was made from 4 representative images/well (n=3 replicates/condition). Cells with uniformly-stained chromatin were scored as healthy whereas those that had chromatin fragmentation or condensation were considered apoptotic.

**Western blot**

Cell homogenates were obtained in RIPA buffer 1 x (ThermoFisher Scientific), supplemented with 1 x EDTA-free complete protease inhibitor cocktail (Roche). 30-50μg of protein were resolved in NuPAGE 4-12% Bis-Tris gels and transferred to iBlot Gel Transfer Stacks PVDF membranes (Life Technologies, Thermo Fisher Scientific). After blocking with Tris-buffered saline with Tween-20 containing 5% non-fat dry milk or 5% bovine serum albumin for 1 hour at room temperature, membranes were incubated overnight at 4ºC with the indicated primary antibody: anti-Caspase-3 (1:1,000 Cell Signaling; #9662), anti-Cleaved CASP3 (1:1,000 Cell Signaling; #9664), anti-Fodrin (1:1,000 Millipore; #MAB1622), anti-E2F2 (1:1,000 Abcam; ab65222), anti-KIF11 (1:1,000[2] ), anti-FADD (1:1,000 Santa Cruz; sc-6035), anti-AKT2 (1:500[3]), anti-CCND1 (1:1,000 Cell Signaling; #2978), anti-MKNK2 (1:100 Sigma-Aldrich; HPA021875), anti-INCENP (1:500[4] ), anti-CHAF1A (1:2,000 Cell Signaling; #5480), anti-BCL-X_L_ (1:10,000 BD Bioscience; #610211), anti-CDC25A (1:500 Thermo Scientific; DCS-120), anti-CCNB1 (1:1,000 Millipore; #05-373), anti-CCNE1 (1:1,000 Abcam; ab3927), anti-p27 (1:1,000 Cell Signaling; #3686), anti-CDK6 (1:1,000 Cell Signaling; #3136), anti-pRB (1:1,000 Cell Signaling; #8516). Membranes were incubated with peroxidase-conjugated secondary antibodies for 1 h with anti-mouse IgG-Perodixase antibody produced in rabbit (1:10,000, Sigma-Aldrich; #A9044) or anti-rabbit IgG-Peroxidase antibody produced in goat (1:10,000, Sigma-Aldrich; #A0545). Anti-actin HRP (1:40,000 Santa Cruz; sc-1616) or α-Tubulin (1:5,000 Sigma-Aldrich; T9026) were used as loading controls. Membranes were developed with a SuperSignal Dura detection kit (Pierce/ThermoFisher Scientific) or EZ-ECL Chemiluminescence detection kit (Biological Industries).

**Quantitative real-time PCR (qPCR)**

Total RNA was extracted using the miRNeasy Mini Kit (Qiagen). mRNA were reverse transcribed (1μg of total RNA) using a Taqman RT kit (Applied Biosystems, Thermo Fisher Scientific). Real-time PCR of the indicated genes was performed using 2X Power SYBR Green Master Mix (Applied Biosystems, Thermo Fisher Scientific). Gene expression was normalized against the *L27* housekeeping gene. Primer sequences are listed in a Supplementary Table 9. Relative quantification of gene expression was performed with a comparative 2^(-ΔΔCT)^ method [5].

***In silico* miRNA-target analysis**

The miRWalk database using five miRNA-target prediction algorithms (DIANA-microT; version 3.0, miRDB, miRWalk[6], miRanda[7] and TargetScan; version 5.1[8]) was used to make the computational miRNA target prediction analysis. The miRNA target search was restricted to the 3’-UTR of target genes and with a minimum complementarity of 7 nucleotides in the seeding region. Probability distribution of random matches was set at 0.05 (Poisson *p*-value). Target genes with *p* ≤ 0.05 predicted by four or all five algorithms were selected as predicted targets. The functional annotations of resulting predicted target lists were performed using the Gene Ontology[9] and the KEGG[10] databases. GSE45547[11] and GSE62564[12] datasets were used for target gene expression analysis in human tumor samples.

**3’ UTR Luciferase Reporter Assay**

The 3’-UTR fragments of *CHAF1A*, *KIF11*, *INCENP*, *CDC25A*, *FADD*, *CCND1* and *BCLX* (Supplementary Table 10) were synthesized by GeneArt Gene Synthesis (Thermo Fisher Scientific) and cloned downstream of the renilla luciferase gene using *Xho*I*/Not*I restriction sites in the psi-CHECK^TM^-2 vector (Promega Corporation). HEK293T cells were seeded in 96-well plates (2 x 10^4^ cells/well) and co-transfected 12-14 h later with 50 ng of psi-CHECK2 reporter vectors and 25 nM of miRIDIAN microRNA mimic oligonucleotides using Lipofectamine 2000. Twenty-four hours later, luciferase activity was measured using the Dual-Glo® Luciferase Assay System (Promega Corporation) following the manufacturer’s recommendations. Luminescence of each biological sample was measured in an Appliskan (Thermo Fisher Scientific) microplate reader. Renilla luciferase activity was normalized to corresponding firefly luciferase activity.

**Supplementary References**

1. Mestdagh P, Fredlund E, Pattyn F, Schulte JH, Muth D, Vermeulen J, Kumps C, Schlierf S, De Preter K, Van Roy N, et al (2010) MYCN/c-MYC-induced microRNAs repress coding gene networks associated with poor outcome in MYCN/c-MYC-activated tumors. Oncogene, 29:1394-1404.

2. Blangy A, Lane HA, d'Hérin P, Harper M, Kress M, Nigg EA (1995) Phosphorylation by p34cdc2 regulates spindle association of human Eg5, a kinesin-related motor essential for bipolar spindle formation in vivo. Cell. Dec 29;83(7):1159-69.

3. Walker KS, Deak M, Paterson A, Hudson K, Cohen P, Alessi DR (1998) Activation of protein kinase B beta and gamma isoforms by insulin in vivo and by 3-phosphoinositide-dependent protein kinase-1 in vitro: comparison with protein kinase B alpha. Biochem J. Apr 1;331 ( Pt 1):299-308.

4. Honda R, Körner R, Nigg EA (2003) Exploring the functional interactions between Aurora B, INCENP, and survivin in mitosis.Mol Biol Cell. Aug;14(8):3325-41. Epub 2003. May 29.

5. Livak KJ, Schmittgen TD (2001) Analysis of relative gene expression data using real-time quantitative PCR and the 2(-Delta Delta C(T)) Method. Methods, **25:**402-408.

6. Vlachos IS, Zagganas K, Paraskevopoulou MD, Georgakilas G, Karagkouni D, Vergoulis T, Dalamagas T, Hatzigeorgiou AG (2015) DIANA-miRPath v3.0: deciphering microRNA function with experimental support. Nucleic Acids Res, 43:W460-466.

7. Betel D, Koppal A, Agius P, Sander C, Leslie C (2010) Comprehensive modeling of microRNA targets predicts functional non-conserved and non-canonical sites. Genome Biol, 11:R90.

8. Agarwal V, Bell GW, Nam JW, Bartel DP (2015) Predicting effective microRNA target sites in mammalian mRNAs. Elife. Aug 12;4. doi: 10.7554/eLife.05005.

9. Ashburner M, Ball CA, Blake JA, Botstein D, Butler H, Cherry JM, Davis AP, Dolinski K, Dwight SS, Eppig JT, et al (2000) Gene ontology: tool for the unification of biology. The Gene Ontology Consortium. Nat Genet, 25:25-29.

10. Kanehisa M, Goto S (2000) KEGG: kyoto encyclopedia of genes and genomes. Nucleic Acids Res 2000, 28:27-30.

11. Kocak H, Ackermann S, Hero B, Kahlert Y, Oberthuer A, Juraeva D, Roels F, Theissen J, Westermann F, Deubzer H, et al (2013) Hox-C9 activates the intrinsic pathway of apoptosis and is associated with spontaneous regression in neuroblastoma. Cell Death Dis 2013, 4:e586.

12. Su Z, Fang H, Hong H, Shi L, Zhang W, Zhang Y, Dong Z, Lancashire LJ, Bessarabova M, Yang X, et al (2014) An investigation of biomarkers derived from legacy microarray data for their utility in the RNA-seq era. Genome Biol, 15:523.

**SUPPLEMENTARY FIGURE LEGENDS**

**Supplementary Figure 1| Experimental design and statistics analysis of the microRNA high-throughput screening.** SK-N-BE(2) cells were transfected with miRNA mimics (25nM) in 96-well plates. **(A)** Control plates were transfected with two mimic miRNA negative controls (control 1: cel-miR-67 and control 2: cel-miR-239b), miR-497-5p as a positive control and a mimic miRNA transfection control with Dy547. Mock-transfected cells were transfected with Lipofectamine without miRNA; rows A and H and columns 1 and 12 were non-transfected (NT) cells. **(B)** MiRNA-mimic plates were transfected with 60 individual miRNA. Rows A and H and columns 1 and 12 were non-transfected (NT) cells. **(C)** Raw absorbance values from three replicates of miRNA mimics plate 2 and the frequency distribution histograms of absorbance values.

**Supplementary Figure 2| MiR-323a-5p and miR-342-5p modulate the expression of multiple cancer-related genes**. (**A,B**) MRNA relative expression levels of predicted miR-323a-5p target genes in SK-N-BE(2) (**A**) and SK-N-AS (**B**) cells transfected with 25 nM of miR-control or miR-323a-5p and analyzed by qPCR. (**C,D**) Relative mRNA expression of miR-342-5p target genes in SK-N-BE(2) (**C**) or SK-N-AS (**D**) cells transfected with 25 nM of miR-control or miR-342-5p mimic oligonucleotides measured by qPCR at 48 h post-transfection. Data represent the mean ± S.E.M. of three independent experiments (n=2 per experiment). **p*<0.05, ***p*<0.01, two-tailed Student’s *t*-test.

**Supplementary Figure 3| MiR-323a-5p and miR-342-5p predicted binding sites for each of the selected target genes.** MiR-323a-5p and miR-342-5p binding sequences within the indicated human genes. Seeding region is flanked by arrows with the indicated nucleotide position within the 3’UTR.

**Supplementary Figure 4|** **Tumor volume correlates with tumor weight.** Correlation between tumor weight and tumor volume in SK-N-BE(2) (**A**) and SK-N-AS xenografts (**B**) transfected with miR-control (left panel), miR-323a-5p (middle panel) or miR-342-5p (right panel).

**Supplementary Figure 5| MiR-323a-5p and miR-342-5p are overexpressed in SK-N-BE(2) and SK-N-AS tumors transfected with each miRNA.** (**A,C**) Relative expression of miR-323a-5p and miR-342-5p in SK-N-BE(2) (**A**) and SK-N-AS (**C**) cells transfected with 25 nM of the indicated miRNA *in vitro* 36 h post-transfection (at the time of mice injection). (**B,D**) Relative expression levels of miR-323a-5p and miR-342-5p in tumor xenografts of SK-N-BE(2) (**B**) and SK-N-AS (**D**) at the end of the experiment. *p<0.05, **p<0.01, ***p<0.001, two-tailed Student’s t-test.


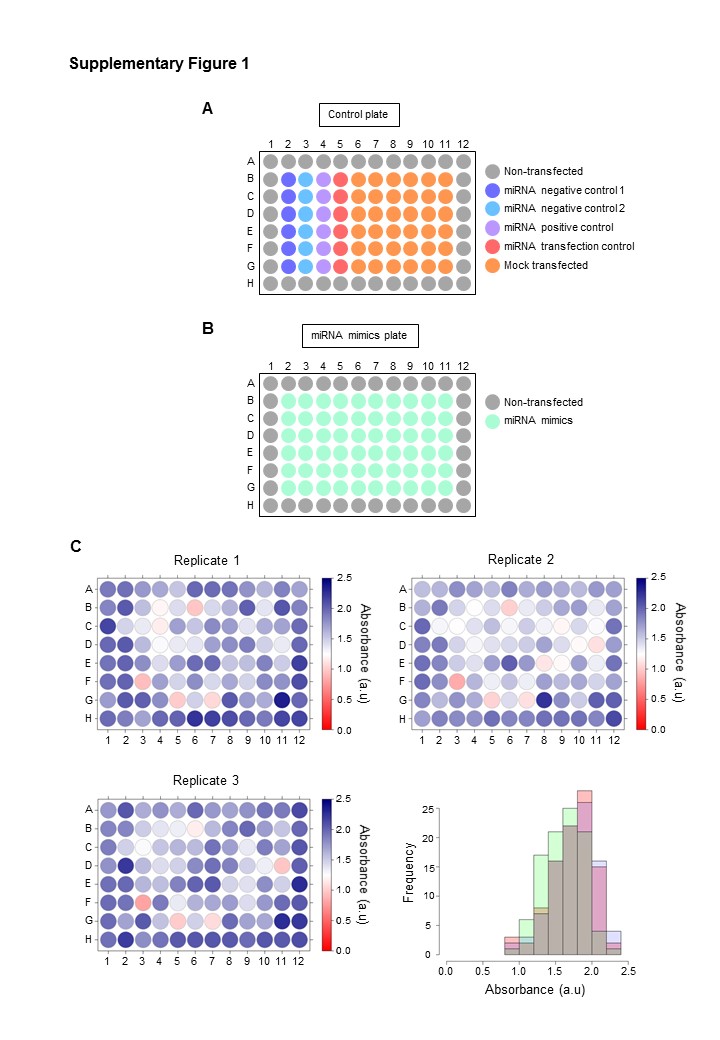


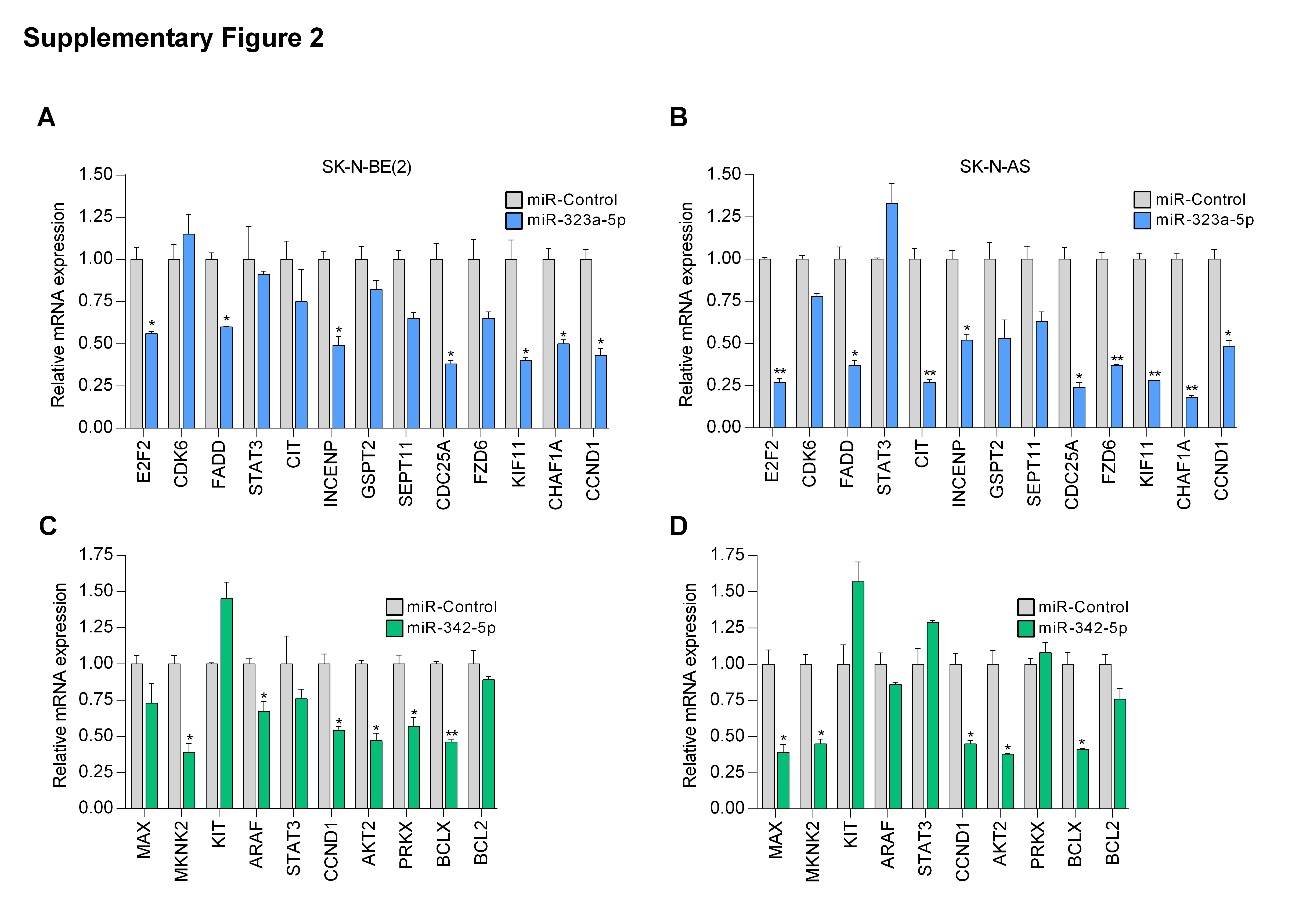


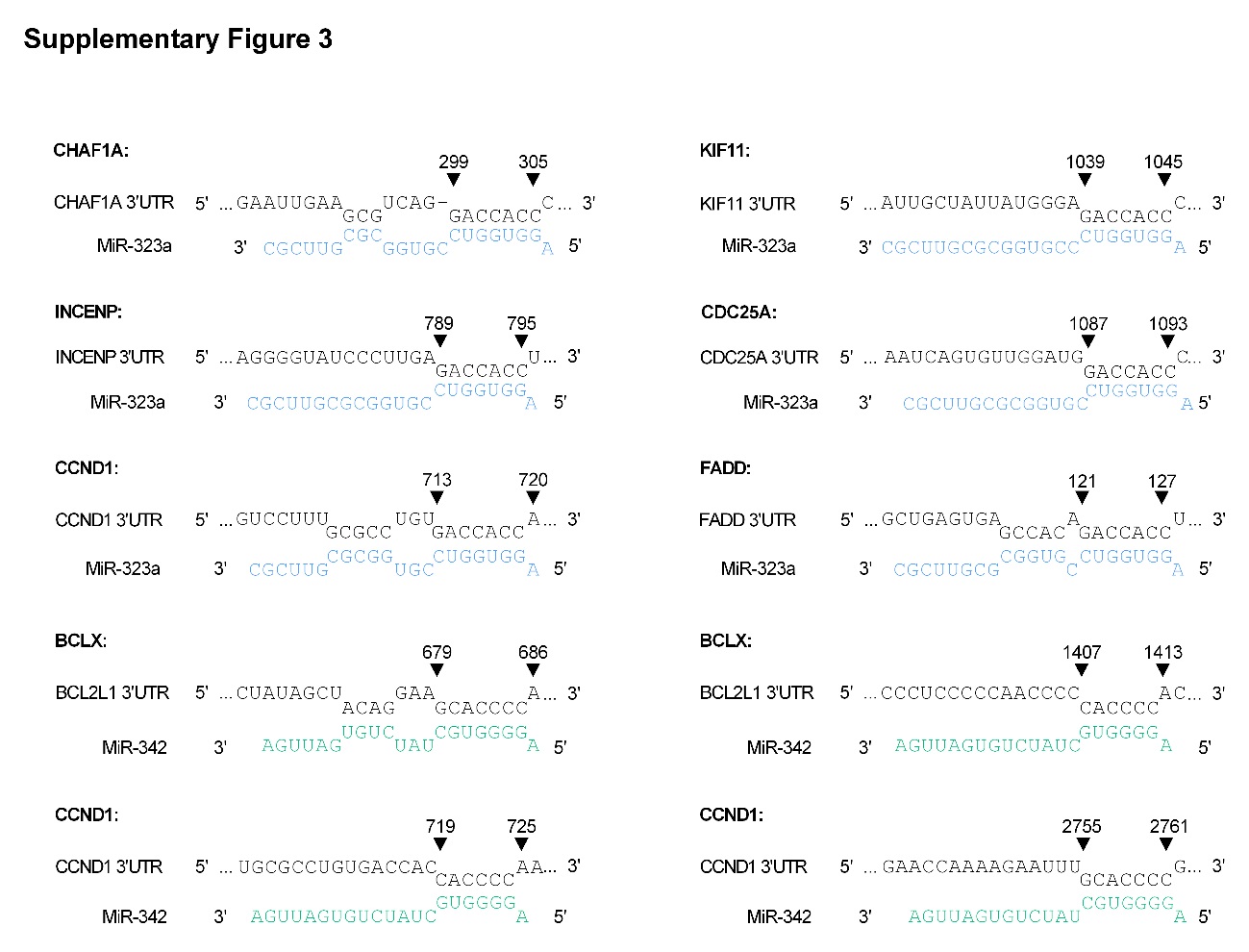

Supplement: Supplementary file 1 — Supplementary material 1 (DOCX 675 kb) [file 18_2019_3041_MOESM1_ESM.docx]
