# Supplementary material for: Functional high-throughput screening reveals miR-323a-5p and miR-342-5p as new tumor-suppressive microRNA for neuroblastoma
Source: Cell Mol Life Sci. 2019 Feb 15;76(11):2231–43. doi: 10.1007/s00018-019-03041-4 (PMC6502783; doi:10.1007/s00018-019-03041-4)
Supplement: Supplementary file 3 — Supplementary material 3 (DOCX 18 kb) [file 18_2019_3041_MOESM3_ESM.docx]

**SUPPLEMENTARY TABLES**

| **Cell lines** | **Stage** | **Age (months)** | **MYCN Status** | **P53 Status** | **ALK Status** | **Chemo response*** |
| --- | --- | --- | --- | --- | --- | --- |
| **CHLA-90** | 4 | 102 | Not amplified | Non-functional | F1245V Mut^1^ | Resistant |
| **SH-SY5Y** | 4 | 48 | Not amplified | Functional | F1174L Mut^1^ | Sensitive |
| **SK-N-AS** | 4 | 72 | Not amplified | Non-functional | NA^2^, wt^3^ | Resistant |
| **LA1-5s** | 4 | 36 | Amplified | Non-functional | F1174L Mut^1^ | Resistant |
| **IMR-32** | 4 | 13 | Amplified | Functional | PA^4^, wt^3^ | Sensitive |
| **SK-N-BE(2)** | 4 | 24 | Amplified | Non-functional | NA^2^, wt^3^ | Resistant |

**Supplementary Table 3**. Neuroblastoma cell lines characteristics.

*DNA damaging agents (e.g. cisplatin, etoposide, etc)

^1^Mut: Mutated; ^2^NA: Not amplified; ^3^wt: wild type; ^4^PA: Partial Amplification.

**Supplementary Table 8.** List of siRNA sequences.

| **Gene** |  | **Sequence (5'-3')** |
| --- | --- | --- |
| **KIF11** | KIF11 | CUAGAUGGCUUUCUCAGUA |
|  | KIF11_as | UACUGAGAAAGCCAUCUAG |
| **INCENP** | INCENP | AGUCCUUUAUUAAGCGCAAUU |
|  | INCENP_as | AAUUGCGCUUAAUAAAGGACU |
| **CHAF1A** | CHAF1A | CUGUCAUGUGGGUUCUGAC |
|  | CHAF1A_as | GUCAGAACCCACAUGACAG |
| **CDC25A** | CDC25A | GGAAAAUGAAGCCUUUGAG |
|  | CDC25A_as | CUCAAAGGCUUCAUUUUCC |
| **FADD** | FADD | UGCGUUCUCCUUCUCUGUG |
|  | FADD_as | CACAGAGAAGGAGAACGCA |
| **CCND1** | CCND1 | CCUACGAUACGCUACUAUAUU |
|  | CCND1_as | AAUAUAGUAGCGUAUCGUAGG |
| **BCLX** | BCLX | CAGGCGACGAGUUUGAACU |
|  | BCLX_as | AGUUCAAACUCGUCGCCUG |

**Supplementary Table 9.** List of qPCR primer sequences.

| **Gene** | **Primer sequence (5' to 3')** | **Amplicon (bp)** |
| --- | --- | --- |
| **KIF11** | Fw: AAAACAACAAAGAAGAGACAATTCC | 93 |
|  | Rv: CAGATGGCTCTTGACTTAGAGGT |  |
| **AKT2** | Fw: CTCACACAGTCACCGAGAGC | 82 |
|  | Rv: TGGGTCTGGAAGGCATACTT |  |
| **CDK6** | Fw: GAACTAGGCAAAGACCTACTTCTGA | 130 |
|  | Rv: GGTGGGAATCCAGGTTTTCT |  |
| **KIT** | Fw: ATGGCATGCTCCAATGTGT | 100 |
|  | Rv: GGCAGTACAGAAGCAGAGCA |  |
| **BCLX** | Fw: AGCCTTGGATCCAGGAGAA | 102 |
|  | Rv: AGCGGTTGAAGCGTTCCT |  |
| **MAX** | Fw: TGTTCCTGGGGAATTCACTT | 99 |
|  | Rv: GACCTCGCCTTCTCCAGTG |  |
| **CHAF1A** | Fw: TCACCCAATTCATGAAGAAGC | 113 |
|  | Rv: GATCATACAGTCGCCCTCCT |  |
| **E2F2** | Fw: AGGGGAAGTGCATCAGAGTG | 84 |
|  | Rv: GCGAAGTGTCATACCGAGTCT |  |
| **SEPT11** | Fw: CGTGGGGAGACCGTCTAA | 106 |
|  | Rv: TCCTTGAGAAGTAGACTTGTTGACC |  |
| **GSPT2** | Fw: TGAGGAATTTACAAACAATGCATAA | 114 |
|  | Rv: TGGACTTGGACAGAGAAAACAA |  |
| **ARAF** | Fw: CATGGTCCAGCTCATCGAC | 119 |
|  | Rv: TGAGCCCCTCATGTAGGAAG |  |
| **MKNK2** | Fw: TGGGCGTCATCTTGTATATCCT | 129 |
|  | Rv: CCTGGATGCTCTCAAACAGC |  |
| **FZD6** | Fw: CGTCTATGAGCAAGTGAACAGG | 114 |
|  | Rv: AATTCTGGTCGAGCTTTTGC |  |
| **PRKX** | Fw: GCAGGAGCAACACGTACACA | 88 |
|  | Rv: GTCATGCCACGTCCAGAAC |  |
| **STAT3** | Fw: CCCTTGGATTGAGAGTCAAGA | 108 |
|  | Rv: AAGCGGCTATACTGCTGGTC |  |
| **CIT** | Fw: ACGTGAAGAAAGTGAAACAGTCC | 130 |
|  | Rv: CAGAGCCCGTTCTCAATCTT |  |
| **INCENP** | Fw: AAGAGCAGCAGCGTCTGG | 124 |
|  | Rv: GAGGTACAAGCTGGAGACTGC |  |
| **FADD** | Fw: AGCGGAGGAGACCAGCTC | 96 |
|  | Rv: ATGGGCTCTGGTGAAGGAT |  |
| **CDC25A** | Fw: CCCAGCTCGGATGCTTTCC | 86 |
|  | Rv: TCACAGGTGACTGGGGTGTA |  |
| **CCND1** | Fw: GCTGCGAAGTGGAAACCATC | 135 |
|  | Rv: CCTCCTTCTGCACACATTTGAA |  |
| **BCL2** | Fw: GGTGGGGTCATGTGTGTGG | 89 |
|  | Rv: CGGTTCAGGTACTCAGTCATCC |  |
| **L27** | Fw: AGCTGTCATCGTGAAGAA | 88 |
|  | Rv: CTTGGCGATCTTCTTCTTGCC |  |

**Supplementary Table 10.** 3’UTR luciferase reporter cloning.

| **MiRNA** | **Gene** | **Fragment of 3’UTR cloned** | **Binding site** |
| --- | --- | --- | --- |
| **MiR-323a** | **CHAF1A_3’UTR** | +1 to +317 | 299-305 |
|  | **KIF11_3’UTR** | +786 to +1226 | 1039-1045 |
|  | **INCENP_3’UTR** | +540 to +1086 | 789-795 |
|  | **CDC25A_3’UTR** | +774 to +1320 | 1087-1093 |
|  | **FADD_3’UTR** | +1 to +546 | 121-127 |
|  | **CCND1(A)_3’UTR** | +642 to +1014 | 713-720 |
| **MiR-342** | **CCND1(A)_3’UTR** | +642 to +1014 | 719-725 |
|  | **CCND1(B)_3’UTR** | +2569 to +2774 | 2755-2761 |
|  | **BCLX_3’UTR** | +618 to +1482 | 679-686 |
|  |  |  | 1407-1413 |
